# The Relationship between Social Support for Physical Activity and Physical Activity across Nine Years in Adults Aged 60–65 Years at Baseline

**DOI:** 10.3390/ijerph20054531

**Published:** 2023-03-03

**Authors:** Genevieve S. E. Smith, Wendy Moyle, Nicola W. Burton

**Affiliations:** 1 School of Applied Psychology, Griffith University, Mt. Gravatt, QLD 4122, Australia; 2 Menzies Health Institute Queensland, Griffith University, Southport, QLD 4215, Australia; 3 Centre for Mental Health, Griffith University, Mt. Gravatt, QLD 4122, Australia; 4 School of Nursing and Midwifery, Griffith University, Nathan, QLD 4111, Australia

**Keywords:** social support, physical activity, older adults

## Abstract

Physical activity is consistently recognized as a key component of healthy aging. The current study aimed to investigate the prospective association between social support specific for physical activity (SSPA) and physical activity across nine years among adults aged 60–65 years at baseline (*n* = 1984). An observational longitudinal design was used, with mail surveys administered to a population-based sample across four waves. SSPA was measured using a score ranging from 5–25, and physical activity was assessed as time spent in walking, or engaging in moderate and vigorous activity, during the previous week. Data were analyzed using linear mixed-effects models. The results demonstrated a positive significant relationship between SSPA and physical activity, accounting for sociodemographic and health variables. Each unit of increase in SSPA was associated with 11 extra minutes of physical activity per week (*p* < 0.001). There was a significant interaction between SSPA and wave at the final timepoint, such that the relationship was weaker (*p* = 0.017). The results highlight the value of even small increases in SSPA. SSPA could be targeted to promote physical activity among older adults, but may be more impactful in young-old adults. More research is needed to understand impactful sources of SSPA, underlying mechanisms between SSPA and physical activity, and potential moderation by age.

## 1. Introduction

The important role of physical activity in healthy aging has been widely documented [1]. Older adults who engage in recommended levels of physical activity have a slower rate of age-related physical decline and a reduced risk of chronic conditions, cognitive decline, and poor psychological health than those who are inactive [2,3]. Physical activity can increase positive psychological wellbeing [4], life satisfaction [5], health-related quality of life [6], confidence, mastery, and self-esteem [7]. Older adults who are physically active use fewer primary health care services and use those services less frequently than inactive older adults [8]. The benefits of physical activity for healthy aging are not limited to those who have been active throughout adulthood—longitudinal data have shown that initiating physical activity in late adulthood also has significant health and wellbeing benefits [9].

The World Health Organization [WHO] Physical Activity Guidelines for older adults (2020) recommend a minimum of 150 min per week of moderate to vigorous aerobic physical activity to provide positive health outcomes [10]. There are marked regional differences in physical activity participation, with prevalence estimates ranging from 20–60% of older adults around the globe achieving WHO guidelines [11]. Population studies have reported that the proportion of adults meeting these guidelines declines with age, with older adults having the lowest prevalence of physical activity among the age groups [12]. The most recent national survey in Australia found that 50% of adults aged 65+ reached the Australian guidelines of 30 min of physical activity at least five days a week [13]. It is important to note, however, that even small amounts of physical activity below the guidelines can provide important health gains and cost savings to governments [14,15]. For example, a systematic review found that in adults 60+ years, half the recommended amount of physical activity (i.e., 75 min/week) led to a 22% reduction in all-cause mortality compared to no activity [16]. Accordingly, more research is needed to understand the factors influencing physical activity among older adults.

Social support is a multidimensional construct broadly defined as emotional and practical assistance underpinning good social relations [17]. It is incorporated in several theories, such as Social Cognitive Theory [18], Self-Determination Theory [19], and the Theory of Triadic Influence [20], as an influence on behavior. Social support may enable physical activity uptake and maintenance via encouragement, resources, and companionship. A systematic review of qualitative studies identified encouragement as a key theme for facilitating physical activity in older adults [21]. Other qualitative studies have identified that resource issues, such as lack of transport and affordability, are key barriers to physical activity in older adults [22]. A cross-sectional, population-based study reported that adults 60+ years who had the company of family and friends during physical activity were more than twice as likely to reach physical activity guidelines than those who did not have company (50% versus 20%) [23]. Similarly, a longitudinal study found that over a 16-week intervention period, those that walked with peers had a greater increase in physical activity than those who walked alone [24]. A mixed-methods review of community-based group physical activity programs for older adults found that social connectedness was a leading influence on long-term activity adherence at 12 months post-program [25]. A longitudinal study found that adults 50+ years who were members of sports or exercise groups had a slower rate of decline in physical activity across 14 years than did the matched controls [26].

A systematic review of the association between social support and physical activity in older adults found no clear findings between general social support and physical activity, but a positive association for social support specific to physical activity [27]. However, nearly all the studies in the review were cross-sectional, thereby limiting the inferences that could be made regarding the direction of the relationship. The authors concluded that prospective studies are needed to further understand the relationship between social support specific to activity and physical activity in older adults [27]. Mixed findings from a review of prospective research on social support and physical activity also highlighted the need to focus on physical activity-specific measures of social support, but focused on young and middle aged subject and not older adults [28]. Therefore, the current study aimed to address these gaps by investigating the prospective association between social support specific for physical activity and physical activity across nine years among older adults (aged 60–65 years at baseline). Based on previous research, it was hypothesized that there would be a positive association.

## 2. Materials and Methods

### 2.1. Study Design

This study used data sourced from HABITAT, an observational, longitudinal, multilevel study of people aged 40–65 years at baseline and conducted in Brisbane, the capital city of Queensland, Australia. A brief overview of the HABITAT study is provided below, with more details available elsewhere [29,30].

### 2.2. Ethics

HABITAT was awarded ethical clearance by The University Human Research Ethics Committee at the Queensland University of Technology (ID3967H). Survey return was taken as informed consent.

### 2.3. Participants and Sampling

Participants in the overall HABITAT study were recruited using a two-stage sampling design, with study areas selected first, followed by the selection of individuals. First census collection districts (CCDs) in Brisbane were ranked into deciles using the Australian Bureau of Statistics (ABS) index of relative socioeconomic disadvantage (ISDR). Then 20 CCDs were randomly selected from each decile to obtain socioeconomic diversity. Within each CCD, a random sample of people aged 40–65 (as the age group of interest) was identified using Australian electoral roll data from March 2007. Potentially eligible participants were invited to the HABITAT study by mail in May 2007. Participants for the current study were those respondents aged 60–65 years (*n* = 1984) at the HABITAT baseline survey.

### 2.4. Procedure

Data were collected from all HABITAT participants using a mail survey method by Dillman [31]. Potential participants initially received a personalized letter explaining the study’s purpose and the importance of their response. The questionnaire was sent a week later with a reply-paid return envelope. After one week, a thank you reminder card was sent. Seven weeks after the initial mail-out, a personalized reminder letter and replacement questionnaire, with a reply-paid return envelope, were sent to non-respondents. A unique participant ID was printed on each questionnaire to enable data matching across survey waves.

Mail surveys were conducted across five waves in 2007, 2009, 2011, 2013, and 2016. Participants were sent the questionnaire at each wave, regardless of whether they had replied to the survey in previous waves. The exception to this was those people who had actively withdrawn, were identified as having died, or could not be contacted because of a change in address. The current study used data from the four survey waves (2007, 2009, 2011, 2016), including the variables of interest.

### 2.5. Measures

#### 2.5.1. Physical Activity

Physical activity was measured at all time points using items from the Active Australia Survey [32]. The items assessed the frequency and duration over the previous week of each of episode of walking (both recreational and for transport), moderate activity (e.g., gentle swimming, social tennis, golf), and vigorous activity (“activity which made you breathe harder or puff and pant, e.g., jogging, cycling, aerobics, competitive tennis)”. Total physical activity time was calculated by summing the time spent in walking, moderate physical activity, and vigorous physical activity, with vigorous activity time doubled due to the higher intensity [32]. Following standardized processes to minimize measurement error from overreporting, each domain of activity was truncated at 840 min/week, and the total time was truncated at 1680 min per week [32]. The Active Australia questions exhibit acceptable reliability and validity and are recommended for Australian population-based research [33]. To describe the current study sample, physical activity was also categorized into meeting guidelines (≥150 weighted min/week) and not meeting guidelines (<150 weighted min/week), which reflects the WHO physical activity guidelines for older adults [10] For the main analysis, physical activity was used as a continuous variable.

#### 2.5.2. Social Support for Physical Activity

Social support for physical activity (SSPA) was measured at all time points using a 5-item scale obtained from previous research [34]. Participants were asked to rate how often family or friends provided each of five different types of SSPA over the last three months, using the response options: 1—Never, to 5—Very often. The items reflected four different types of support: emotional: “encouraged you to do physical activity”, instrumental: “done something to help you be physically active”, informational: “discussed physical activity with you”, and companionship: “done or offered to do physical activity with you” and “invited you to do physical activity with them”. Responses for these five items were summed to create total SSPA scores ranging from 5–25, with higher scores indicating more support. Previous research has shown that the SSPA items load in a single factor which support the scales use as a unidimensional measure [34]. The internal reliability of the 5-item scale in the current study using the 2007 data (*n* = 1984) was α = 0.91.

#### 2.5.3. Sociodemographic and Health Measures

The following constructs were assessed at all time points and used in the current study: gender (male/female), date of birth (month/years), country of birth (Australia/other specified), living arrangement (alone no children/single parent living with one or more children/single living with friends or relatives/couple living with no children/couple living with one or more child/other), employment status (full-time work/part-time work/casual work/work without pay/home duties not looking for work/unemployed looking for work/retired/permanently unable to work/student/other). Self-rated health was measured using a single item asking, “In general, how would you describe your health?” with responses recorded on a Likert scale of 1—poor, to 5—excellent. Highest educational qualification (less than year 12/year 12/trade certificate, apprenticeship, diploma, certificate/bachelor’s degree/masters or doctorate) was assessed only at baseline. The baseline data were used to describe the current study sample in the demographics table.

#### 2.5.4. Data Analysis

Data were analyzed in R statistical program version 4.0.5 [35]. Descriptive data were produced using R package psych [36], and the figure was created using ggplot2 [37]. Linear mixed-effects models were fitted and analyzed using lme4 [38], and *p*-values were calculated using lmerTest [39]. A Satterthwaite adjustment was used to compute the degrees of freedom. A step-by-step approach was used to build the final model [40], starting with a simple model and then adding additional effects one at a time, checking whether the addition increased the model fit between steps. Model fit was tested using the Akaike information criterion (AIC) (see Table S1 in the Supplementary Materials for each step and AIC values), where a lower AIC value indicates a better quality of fit relative to the complexity of the model [41]. The base model included SSPA and wave as fixed effects and the participant as a random effect to account for the probable non-independence of observations from the same participant. In the second step, an interaction effect between the social support and the wave was added to investigate if the effect of social support on physical activity differed across time. Subsequently, covariables were added one at a time as fixed effects and included gender, education, employment status, living arrangement, and self-rated health. These covariates were selected based on prior research demonstrating an association with physical activity [42]. Graphical assumption testing was completed using sjplot [43]. This indicated some issues regarding the homoskedasticity assumption. In response, a log transformation of the physical activity variable was performed. However, the transformed data created a less desirable distribution and was seen as less interpretable. Therefore, the untransformed data was retained. Research has shown that mixed-effects models are robust to assumption violation, resulting in minimal overall bias [44].

## 3. Results

### 3.1. Participant Characteristics

**Figure 1:**
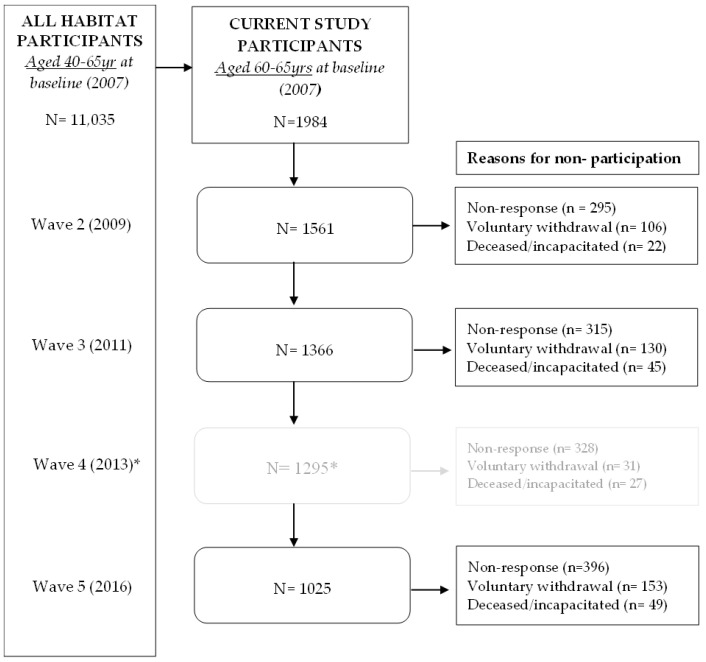
Flow of HABITAT participants aged 60–65 years at baseline over the 9 years of study. Note: Non-response are participants who did not return a survey or were not available at that time point (e.g., travelling overseas), but may have responded at subsequent time points. * Wave 4 (2013) was not used in this analysis, as social support for physical activity (SSPA) was not measured at this time point. Participant numbers are provided to describe the flow from Wave 3 to 5 and the derivation of the final analytic sample.

Participants were 1984 community-dwelling adults aged 60–65 years at the baseline survey. Participants had a mean (standard deviation) age of 61.7 (1.8) years. Two-thirds lived as a couple (65%), half were currently working (49%), half had completed post-school qualifications (49%), 40% reported excellent/very good health, and 57% met WHO physical activity guidelines. See Table 1 for further sociodemographic details on the current study sample. The flow of participants throughout the four-time points of this study can be seen in Figure 1.

### 3.2. Descriptive Results

**Figure 2:**
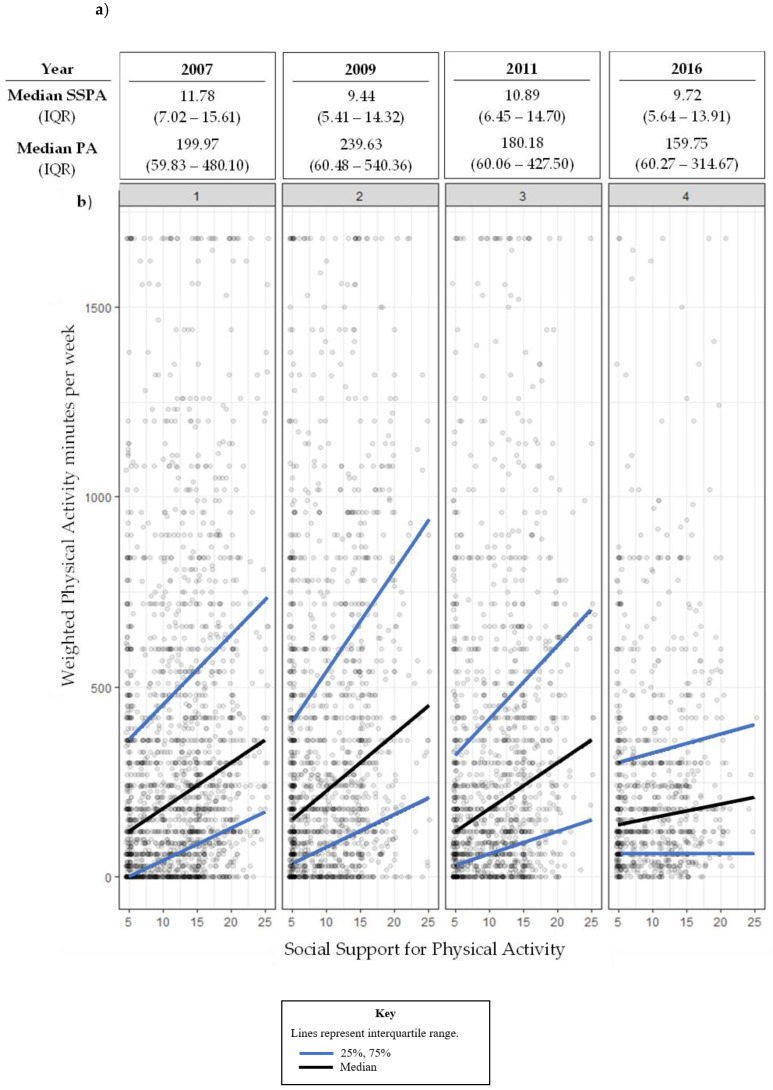
Relationship between social support for physical activity (SSPA) and physical activity (PA) at each wave: (**a**) overall median (IQR); (**b**) scatterplot of SSPA and PA scores.

Figure 2 represents the relationship between SSPA and physical activity at each wave. Median and interquartile values were used, given the skewed distribution. The overall median and interquartile range values at each wave are presented in Figure 2a. In the Figure 2b scatterplots, each dot represents the SSPA and physical activity raw scores for one participant at that wave. The black line connects the median values and reflects the direction and strength of the relationship between the two variables, with the blue lines indicating the interquartile range. The plots suggest a positive relationship between the two variables, with a weaker relationship noted in 2016.

### 3.3. The Relationship between Social Support for Physical Activity (SSPA) and Physical Activity over Time

The results from the linear mixed-effects model (see Table 2) indicated a significant positive relationship between SSPA and physical activity, with each unit increase of SSPA equating to an increase of 11 min per week of physical activity (*p* < 0.001). However, the interaction effect was only significant at wave four, with SSPA having a lower impact on physical activity at wave four than wave one (5 min per week physical activity increase per 1 unit of SSPA increase, versus 11 min per week physical activity increase per 1 unit of SSPA increase respectively, *p* < 0.001).

## 4. Discussion

This study aimed to explore the longitudinal relationship between social support for physical activity (SSPA) and physical activity in adults aged 60–65 years at baseline. This addresses the knowledge gap of prospective evidence on social support specific to physical activity. Overall, a significant positive relationship was found, with each unit of increase in SSPA associated with 11 extra minutes of physical activity per week. This relationship was maintained across the nine years of the study; however, at the last wave, the relationship weakened with each unit of increase in SSPA associated with only five extra minutes of physical activity per week. These findings improve the understanding of social support and physical activity in older adults and can be used to inform directions for practice and future research.

Overall, there was a significant positive relationship between SSPA and physical activity. This is consistent with the findings from a previous review that included cross-sectional studies looking at SSPA in older adults [27]. These results also align with other physical activity studies that highlight the importance of social factors, including a longitudinal study on sport/exercise group membership [26] and a review of qualitative research on community group programs [25]. In the current study, each unit increase in SSPA (on a scale between 5–25) was associated with an additional 11 min of physical activity per week. An example of a one-unit increase in social support is the frequency of emotional, instrumental, or companionship support changing from none to rarely, or from rarely to sometimes, over the last month. This combined evidence demonstrates that SSPA positively influences physical activity, with the current study demonstrating that even small changes in support can make a difference.

The interaction between SSPA and survey wave (which can be seen as a proxy for age) was only significant at the final time point, where the average age of the participants was 71 years. In wave four, although there was still a significant and positive relationship between SSPA and physical activity, the strength of SSPA influence was 55% less than in wave one. One explanation for this weaker association may be that other factors influence physical activity more strongly with increasing age. One such potential influence is declining health. Physical limitations due to health conditions are a leading barrier to physical activity in older adults [45], and health-related problems increase with age [46]. In the current study, the covariate of self-rated health had the strongest relationship with physical activity; those who reported fair/poor health performed 204 min/week less physical activity than those with very good/excellent health. Self-efficacy has been shown to mediate between social support and self-care behavior in older adults with chronic pain [47], so it may be that as health declines, self-efficacy for physical activity declines, thereby reducing the influence of SSPA on physical activity.

The methods and findings from the current study have several implications for practice and research. SSPA may be a useful inclusion in the design of physical activity interventions for adults 60+, with even small increases in SSPA associated with increased physical activity. SSPA elements can include buddy-systems [48], peer-led activities [49], and group-based physical activity [50], such as community-based walking groups [51]. Such interventions may be more successful in promoting physical activity among young older adults, given the results of the current study, which show that SSPA had a weaker influence on physical activity in the fourth wave (when participants were aged 69–74). More research is needed to understand how age moderates the availability and impact of SSPA.

In the current study, SSPA items only asked about support from family and friends. Therefore, participants may not have considered additional support sources, such as medical professionals or other experts [52]. General practitioners, health workers, and community support workers can also have a key role in promoting physical activity [53]. Previous research found that primary care settings are the most trusted source of physical activity information, particularly for older adults, people with chronic diseases, and those who are insufficiently active [54]. Future research could explore the different sources of SSPA across time in older adults. Social support from family members, for example, has previously been reported as particularly important for physical activity among older adults [27]. The SSPA measure combined different types of social support, and future research could explore the different types of SSPA (emotional, instrumental, informational, companionship) and their relative relationships with physical activity overall, as well as with different types and intensities of activity (e.g., walking vs vigorous activity). Future research could also look at factors that may impact upon SSPA in older adults, such as changes in employment status, living arrangements, or health. Other research has, for example, demonstrated that family and friends may be overprotective against physical activity among people with heart failure [55].

A strength of this study is the mixed-effects longitudinal design. This provided the analysis the power to provide evidence on the direction of the association between SSPA and physical activity, as well as to observe the magnitude of the association across time. Another strength is that the measure used captured social support specific to physical activity. A previous review highlighted the need for this specificity [28], with the authors reporting that research often does not assess social support in the comprehensive manner it requires. This is also consistent with ecological models of behavior, which posit the need for behavior specificity [56]. Another potential direction for future research could be to study the relationship between general social support and SSPA. It may be that there is a strong correlation between these two, or it could be that general social support does not always encompass SSPA. Physical activity was reported in minutes per week, and not as guideline attainment, which allowed us to quantify the impact of SSPA and capture changes in physical activity below guideline attainment. The large sample allowed us to control for multiple covariates, including gender, education, living arrangements, employment status, and self-rated health.

However, this study has several limitations. Self-report data were used, which are vulnerable to social desirability and recall bias. Given the mail survey approach, participants were required to subjectively interpret questionnaire items and had no opportunity for familiarization or interpretation clarification. Participants were located in one Australian capital city, and only young older adults were included, with the upper age limit of participants being 75 years after nine years. Therefore, the results may not generalize to rural and non-Western populations or older adults aged 75+. Data were sourced from an overarching study of health and recreation, which may have attracted only participants interested in this topic. A total of 57% of participants in the current study were classified as meeting physical activity recommendations. Therefore, different results may have been obtained with a less active sample.

## 5. Conclusions

The current study demonstrated a significant positive relationship between SSPA and physical activity in adults 60–65 years at baseline. The relationship between SSPA and physical activity was present across the nine years of the study, although the relationship weakened at the last wave. These results suggest that SSPA from friends or family could be included in physical activity interventions for older adults, but may be more effective for older adults aged 60–70, rather than older adults aged 70–75. More longitudinal research with older adults is needed to understand the interaction between age and SSPA, as well as the effect of different types and sources of social support across time, including support from professionals, factors impacting on SSPA, the relative importance of different components of SSPA, and the association between SSPA and general social support. At a population level, improved physical activity among older adults will increase wellbeing, engagement, and productivity and reduce the strain on government resources predicted with an aging population.

## Figures and Tables

**Table 1:** Summary demographics of current study sample at baseline, by gender (*n* = 1984).

	Men *n* = 761	Women *n* = 1223	Total *n* = 1984
	M (SD)	M (SD)	M (SD)
Age (years)	61.69 (1.80)	61.63 (1.77)	61.66 (1.77)
	*n* (%)	*n* (%)	*n* (%)
Country of birth			
Australia	547 (71.9)	928 (76.1)	1475 (74.5)
Other Country	213 (28.0)	292 (23.9)	505 (25.5)
Education completed			
Year 10 or less ^a^	200 (26.3)	547 (44.8)	747 (37.9)
Year 11 or 12 ^b^	86 (11.3)	167 (13.7)	253 (12.8)
Certificate/diploma ^c^	266 (35.0)	168 (22.0)	534 (27.1)
Bachelor’s degree or higher ^d^	204 (26.8)	235 (19.3)	439 (22.0)
Employment status			
In paid workforce ^e^	442 (58.2)	521 (42.6)	963 (48.5)
Not in paid workforce ^f^	318 (41.8)	700 (57.2)	1018 (51.3)
Living arrangement			
Living alone	180 (23.7)	278 (22.7)	458 (23.1)
Single, living with a relative or friend ^g^	71 (9.3)	142 (11.6)	213 (10.7)
Couple, living with no children	394 (51.8)	648 (53.0)	1042 (52.5)
Couple, living with children	106 (13.9)	139 (11.4)	245 (12.3)
Other	1 (0.1)	5 (0.4)	6 (0.3)
Self-rated health			
Excellent/Very Good	311 (40.9)	479 (39.2)	799 (40.2)
Good	291 (38.2)	481 (39.3)	772 (38.9)
Fair/Poor	154 (20.2)	252 (20.6)	406 (20.5)
Physical activity			
Meeting Guidelines	440 (57.8)	694 (56.7)	1134 (57.2)
Not Meeting Guidelines *	295 (38.8)	485 (39.7)	780 (39.3)

Note: Collapsed response categories include: education: ^a^ year 9, year 10; ^b^ year 11, year 12; ^c^ trade certificate, apprenticeship, diploma, certificate ^d^ bachelor degree; Master’s degree or Doctorate. Employment status includes: ^e^ full-time, part-time, and casual work status. Not in the paid workforce includes: ^f^ those working without pay, home duties, unemployed, students, and others. Living Arrangements include: ^g^ single parent living with one or more children, single living with friends or relatives. * Based on WHO physical activity guidelines.

**Table 2:** Associations between physical activity and social support for physical activity (SSPA) over 9 years.

Parameter	Estimate	95% CI	Test Statistic (df)	*p*
Fixed Effects				
(Intercept)	291.16	239.19–343.13	*t* = 10.98 (4162)	<0.001
SSPA Total	11.02	8.17–13.87	*t =* 7.58 (5012)	<0.001
Survey wave				
2007	ref			
2009	33.35	−18.64–85.34	*t* = 1.26 (4034)	0.209
2011	−3.51	−57.76–50.74	*t =* −0.13 (4010)	0.889
2016	−55.90	−117.77–5.97	*t =* −1.77 (4107)	0.077
Gender				
Male	ref			
Female	−66.54	−93.36–−39.71	*t =* −4.86 (1796)	<0.001
Education completed				
Year 10 or less	ref			
Year 11 or 12	35.20	−6.52–76.91	*t* = 1.64 (1875)	0.098
Certificate or diploma	41.73	8.88–74.58	*t* = 2.49 (1823)	0.013
Bachelor’s degree or higher	67.06	32.40–101.72	*t* = 3.79 (1845)	<0.001
Living arrangement				
Living alone	ref			
Living with others	−47.29	−73.74–−20.83	*t* = −3.50 (3471)	<0.001
Employment status				
In paid employment	ref			
Not in paid employment	83.26	60.61–105.90	*t* = 7.21 (4904)	<0.001
Self-rated health				
Excellent/very good	ref			
Good	−98.51	−120.65–−76.38	*t* = −8.72 (5098)	<0.001
Fair/poor	−204.12	−233.49–−174.76	*t* = −13.63 (4582)	<0.001
SSPA Total * Wave				
2007	ref			
2009	2.40	−1.91–6.71	*t* = 1.09 (4072)	0.275
2011	−2.68	−7.05–1.70	*t* = −1.20 (4027)	0.230
2016	−6.33	−11.54–−1.12	*t* = −2.38 (4081)	0.017
Random effects				
ICC	0.38			
N id	1925			
Observations	5125			

Note: SSPA total on a scale of 5–25, with higher scores indicating more support. * = interaction.

## Data Availability

For researchers interested in collaborating on data analyses with the chief investigators, inquiries can be submitted to HABITAT Lead Investigator, Gavin Turrell (gavin.turrell@rmit.edu.au).

## Supplementary Materials

The following supporting information can be downloaded at: https://www.mdpi.com/article/10.3390/ijerph20054531/s1, Table S1: Akaike Information Criterion (AIC) values for linear mixed-model building.
