# Supplementary material for: The Relationship between Social Support for Physical Activity and Physical Activity across Nine Years in Adults Aged 60–65 Years at Baseline
Source: Int J Environ Res Public Health. 2023 Mar 3;20(5):4531. doi: 10.3390/ijerph20054531 (PMC10002128; doi:10.3390/ijerph20054531)
Supplement: Supplementary file 1 [file ijerph-20-04531-s001.zip › ijerph-2112236-supplementary.pdf]

Supplementary materials

Table S1. Akaike Information Criterion (AIC) values for linear mixed model build

Model	Fixed effects	Random Effect	AIC
**1**	SSPA[1]	participant	74506
**2**	SSPA[1] + wave	participant	74369
**3**	SSPA[1] + wave + SSPA*wave	participant	74365
**4**	SSPA[1] + wave + SSPA*wave + gender	participant	74534
**5**	SSPA[1] + wave + SSPA*wave + gender + education	participant	74330
**6**	SSPA[1] + wave + SSPA*wave + gender + education + employment	participant	74303
**7**	SSPA[1] + wave + SSPA*wave + gender + education + employment + health rating	participant	74123
**8**	SSPA[1] + wave + SSPA*wave + gender + education + employment + health rating + living arrangement	participant	74113

Note: A lower AIC value indicates better quality of fit relative to the complexity of the model; therefore, the model with the lowest AIC was selected.
[1]SSPA = Social support for Physical Activity
